# The effect of 14 days Actovegin administration with or without high intensity training on exercise capacity and skeletal muscle mitochondrial respiration

**DOI:** 10.1007/s00421-025-06118-0

**Published:** 2026-01-19

**Authors:** Rasmus Kinimond Hassø, Søren Lindtofte, Brandon Kosik, Andreas Bergdahl, Steen Larsen

**Affiliations:** 1 Department of Biomedical Sciences, Faculty of Health Sciences, University of Copenhagen, 2200 Copenhagen, Denmark; 2 Department of Health, Kinesiology and Applied Physiology, Concordia University, Montreal, Canada; 3 Clinical Research Centre, Medical University of Bialystok, Bialystok, Poland

**Keywords:** High intensity exercise, Performance, Mitochondria, Ergogenic effect

## Abstract

**Purpose:**

Evidence exists that Actovegin has enhancing effects on mitochondrial respiratory capacity (MRC) in skeletal muscle, and it is well known that high intensity training (HIT) improves MRC and exercise performance. The aim was to investigate the effects of Actovegin administration on exercise performance and mitochondrial respiratory capacity in mice skeletal muscle fibers alone or in combination with HIT.

**Methods:**

40 healthy male mice were randomized into 4 groups; control (C), Actovegin (A), trained control (CT) and trained Actovegin (AT). All mice were given intraperitoneal injections every other day for 14 days of either 0.1 ml Actovegin (10 mg/ml) or 0.1 ml saline. The training consisted of a HIT protocol (5x1 min at 80-90 % of maximal running speed with 2 minutes of active recovery in between) performed every other day by the trained groups. All mice completed a maximal exercise capacity test before and after the intervention. High-resolution respirometry was used to measure skeletal muscle mitochondrial respiratory capacity. Citrate synthase activity was used as a biomarker for mitochondrial content.

**Results:**

The CT and AT groups improved their exercise capacity significantly after the 14-day intervention compared to C and A. The increase in performance was significantly higher in the AT group compared to the CT group. Complex I + II linked mitochondrial respiratory capacity was increased in the A, CT and AT groups compared to the C group.

**Conclusion:**

This is the first study showing enhancing effects of Actovegin on exercise performance and skeletal muscle mitochondrial respiratory capacity with in vivo administration in mice.

**Graphical abstract:**

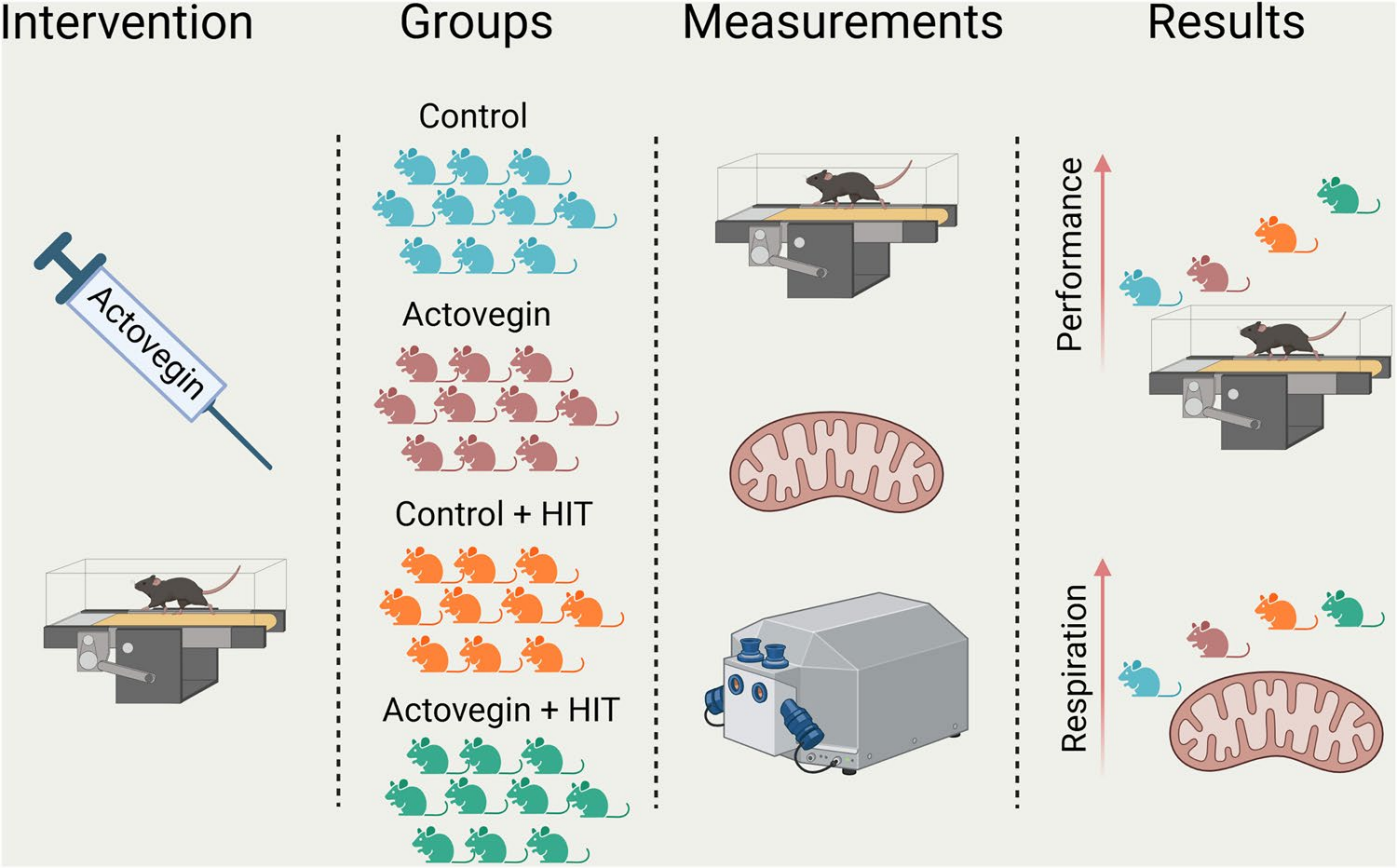

## Introduction

Actovegin is an ultrafiltrate derived through hemodialysis of calf blood containing more than 200 physiological compounds. The filtration process results in a molecular weight of less than 5000 Daltons, meaning Actovegin is free of proteins and hormones (Buchmayer et al. 2011). However, Actovegin contains a wide array of vitamins, minerals and amino acids as well as lactate, succinate and inositol-phospho-oligosaccharides (Machicao et al. 2012; Reichl et al. 2017). Actovegin, known since the 1960s, has been used for numerous therapeutic treatments, including muscle injuries (Lee et al. 2011b), wounds, burns and skin disorders (Buchmayer et al. 2011), radiation-induced damages (Beetz et al. 1996) and acute ischemic stroke (Derev’yannykh et al. 2008). Actovegin has also been shown to have insulin-like effects and to increase oxygen uptake and utilization during hypoxia (Machicao et al. 2012), leading to speculations that it may induce ergogenic effects. These speculations came to light in 2000, after the judicial authorities of France initiated an investigation regarding the use of Actovegin by the cycling team US Postal Service following their Tour de France victory in 2000. The investigation led to the IOC banning the use of Actovegin in December 2000 on the basis that Actovegin was believed to improve oxygen transport similarly to erythropoietin (EPO) (Tsitsimpikou et al. 2009). However, the ban was revoked just two months later due to the lack of scientific evidence. Consequently, Actovegin is still permitted for athletes today, as long as intravenous administration does not exceed 100 ml per 12 h, as is the general guidelines from the World Anti-Doping Agency regarding all intravenous administrations (WADA 2023).

### Potential ergogenic effects of Actovegin

Only a few studies have examined Actovegin from an ergogenic perspective. One study investigated the effect of 40 ml of intravenously injected Actovegin in 8 male participants 2 h prior to exhaustive arm crank exercise when compared to saline injections, no differences were found for Watt-peak, VO_2_-peak, or respiratory compensation point (Lee et al. 2012). Interestingly, 4 of the 8 participants subjectively reported that they found the exhaustive arm crank test easier after Actovegin injection compared to placebo, which the authors explained with Actovegin having a potential effect on attenuating peripheral muscle fatigue. However, the authors concluded that Actovegin has no ergogenic effects and that it is unlikely to enhance performance.

One study examined the acute effects of Actovegin on mitochondrial respiratory capacity (MRC) in permeabilized human skeletal muscle fibers ex vivo using high-resolution respirometry (HRR) (Sondergard et al. 2016). The authors observed an increase in MRC in a concentration-dependent manner when measured as both complex I + II stimulated respiration and the total capacity of the electron transfer system. The measured increases in MRC were attributed to Actovegin having an enhancing effect on the intrinsic function of the mitochondria, since mitochondrial content would not be changed in this acute setting. However, whether these ex vivo results translate to an actual in vivo effect and meaningful improvements in exercise performance is yet to be answered.

### Mitochondrial function and exercise capacity

The energy required to maintain continuous aerobic exercise comes mainly from the ATP molecules generated through mitochondrial oxidative phosphorylation (OXPHOS). Although central factors such as cardiac output and total hemoglobin mass are the limiting parameters regarding maximal aerobic exercise capacity (Bassett and Howley 2000), peripheral factors such as mitochondrial function cannot be overlooked. Increased MRC contributes to increased fat oxidation, resulting in less depletion of glycogen, as well as to decreased accumulation of local fatigue-inducing factors (Holloszy and Coyle 1984). These aspects all contribute to increased exercise capacity, and one study even suggests skeletal muscle MRC may be the best predictor for endurance exercise performance (Jacobs et al. 2011). Exercise training is a strong stimulus for mitochondrial biogenesis, and as few as six sessions (8–12 times 60 s at 100% peak power with 75 s recovery) of high intensity interval training (HIIT) have been shown to improve exercise capacity as a result of improved skeletal muscle MRC (Jacobs et al. 2013b).

### Study aim and hypothesis

The aim of this study was to investigate the effects of a 14-day Actovegin administration on exercise performance with and without HIT. In relation to this, skeletal muscle MRC and citrate synthase activity (used as a biomarker for mitochondrial content) was assessed. Our hypothesis was that Actovegin would increase exercise performance and that we would see an additive effect when combined with HIT, which also would be seen in mitochondrial respiratory capacity.

## Methods

All experimental procedures were carried out at the Department of Health, Kinesiology & Applied Physiology at Concordia University, Montreal, CA. The Animal Ethics Committee of Concordia University approved the study (ID: 30000259). All procedures were conducted in accordance with guidelines of the Canadian Council on Animal Care.

### Animals

The mice (C57BL/6, *N* = 40) were 38 ± 1 weeks old males with a weight of 38 ± 1 g upon inclusion. The age window of the mice was picked to provide a practical and biologically meaningful model for understanding how exercise influences adult physiology and how early-life interventions might shift long-term health trajectories. The mice were kept in individual cages, with unlimited chow diet and water supply, in a temperature-controlled room with a 12-hour light/dark cycle. Every mouse completed a treadmill inclusion test for familiarization purposes. The mice were randomized into 4 groups (*N* = 10 in each group); control (C), Actovegin (A), trained control (CT) and trained Actovegin (AT). All mice were given intraperitoneal injections every other day for 14 days of either 0.1 ml Actovegin (10 mg/ml) or 0.1 ml saline solution. Body weight was also registered every other day. The mice were euthanized by CO_2_ overdose, followed by cervical dislocation.

### Exercise performance test and exercise intervention

All mice were going through a familiarization running test prior to the exercise capacity test on day 0, to make sure that the mice were interested in running. Ensuring that exhaustion was identified consistently and humanely was a central priority in our study. To do this, we relied on well-established behavioral criteria that are widely used in rodent treadmill protocols (Dougherty et al. 2016; Castro and Kuang 2017). Specifically, exhaustion was defined as the point at which a mouse could no longer maintain its position on the treadmill belt despite repeated gentle encouragement and failed to resume running after three consecutive prompts. At this stage, the animal consistently drifted to the back of the treadmill and was unable to recover its gait or re-engage with the pace of the belt. The same trained researcher monitored all exercise sessions to ensure uniform interpretation of these behavioral criteria. On experimental day 0 and 14 the mice performed a maximal exercise capacity test until exhaustion. The test was done with a 10-degree incline on a multi-lane treadmill specifically designed for mice (Bouganim and Bergdahl 2017). The exercise capacity test consisted of a 3-minute warmup at a speed of 13.3 m/min, with a speed increase of 3.3 m/min until exhaustion. After completion, maximal running speed (m/min) was noted for each mouse. The two trained groups performed a HIT protocol specifically designed for mice (Caru et al. 2019) every other day although as a modified version. The HIT training was conducted on the following days (1, 3, 5, 7, 9, 11, 13) in the afternoon giving a total of 7 training sessions. The HIT protocol consisted of a 5-minute warmup at a speed of 13.3 m/min. Afterwards, 5 intervals of one minute duration were performed, with an intensity of 80–90% of maximal running speed obtained in the pre-intervention exercise capacity test (precise intensities were calculated to 84.0% for the CT group and 85.7% for the AT group). In between intervals 2 min of active recovery were performed at 13.3 m/min (60–65% of maximal running speed). The HIT protocol ended with a 5-minute cooldown at 13.3 m/min.

Injections with Actovegin or saline and the exercise training was not blinded for the researchers conducting the experiments. The injections were given on the training days but in the morning where the animals weight was measured as well, giving a total of 7 injections of either Actovegin or saline.

### Tissue preparation and preservation

All mice were euthanized 24 h after completion of the post intervention maximal exercise capacity test, to avoid the acute effect of exercise. Immediately after euthanasia the mice were dissected and both vastus lateralis muscles were extracted and put in physiological saline solution (NaCl 135.5 mM, KCl 5.9 mM, MgCl 1.2 mM, glucose 11.6 mM, HEPES 11.6 mM, pH 7.35). One of the vastus lateralis was preserved at -80 °C for subsequent enzyme analysis (always the left), while the opposite vastus lateralis was kept on ice and prepared for HRR analysis. The muscle fibers were dissected into smaller bundles using two needles and subsequently put into 3 ml of BIOPS (CaK_2_EGTA 2.77 mM, K_2_EGTA 7.23 mM, Na_2_ATP 5.77 mM, MgCl_2_⋅6H_2_O 6.56 mM, Taurine 20 mM, Na_2_Phosphocreatine 15 mM, Imidazole 20 mM, Dithiothreitol 0.5 mM, MES 50 mM, pH 7.1) and 30 µl saponin (5 mg/ml - Sigma S7900) for 30 min. Afterwards the muscle tissue was washed twice for 10 min in 5 ml of MiR05 (EGTA 0.5 mM, MgCl_2_⋅6H_2_O 3.0 mM, K-lactobionate 60 mM, Taurine 20 mM, KH_2_PO_4_ 10 mM, HEPES 20 mM, Sucrose 110 mM, BSA 1 g/L, pH 7.1). The vastus lateralis muscle was examined, because it contains both slow- and fast twitch muscle fibers, and it has been shown to be suitable when progressing from descriptive to inferential statistics between mouse and human mitochondria (Jacobs et al. 2013a).

### Mitochondrial measurements

Mitochondrial respiratory capacity was assessed using HRR (Oxygraph-2k, Oroboros Instruments, Innsbruck, Austria). The titration protocol included malate (1 mM) and glutamate (10 mM) for complex l linked leak assessment. Cytochrome C (10 µM) was added to test the integrity of the outer mitochondrial membrane. If respiration increased more than 10% after addition of cytochrome C the data was removed from the analysis. An ADP-titration followed (0.025–0.05–0.1–0.25–0.5 mM) with the addition of MgCl (3 mM) for assessment of complex l linked respiration. Succinate (10 mM) was added to assess complex l + ll linked respiration. All experiments were carried out at 37 °C at an oxygen range between 200 and 450 nmol/L to ensure oxygen was not a limiting factor. Mitochondrial content was assessed using citrate synthase (CS) activity as biomarker (Larsen et al. 2012). CS activity specific flux, also known as mitochondrial intrinsic respiratory capacity, was calculated as mass specific MRC divided by CS activity (biomarker for mitochondrial content). Data was analyzed using Oroboros DatLab version 6.1.0.7.

### Enzyme analysis

CS activity was measured using spectrophotometry. Skeletal muscle tissue was homogenized in 600 µl 0.3 M K_2_HPO_4_, 0.05% bovine serum albumin (BSA) (pH 7.7) for 2 min on a Tissuelyzer (Qiagen, Venlo, Limburg, Netherlands). 6 µl of 10% triton was added and the samples were left on ice for 15 min. The homogenate was diluted 50 times in a solution containing 0.33 mM acetyl-CoA, 0.6 mM oxaloacetate, 0.157 mM 5,5’-dithiobis-(2-nitrobenzoic acid) (DTNB), 39 mM Tris-HCl (pH 8.0). The change in DTNB to TNB at 37 °C was measured spectrophotometrically at 415 nm on an automatic analyzer, Cobas 6000, C 501 (Roche Diagnostics, Mannheim, Germany). CS activity is expressed as micromoles substrate per minute per gram dry weight of tissue.

### Statistical analysis

Data was analyzed using ANOVA and linear mixed effects models, with Tukey’s multiple comparisons test post-hoc. Normality was assessed using the Shapiro-Wilk test and by interpreting QQ and residual plots. Citrate synthase activity was analyzed using an ANOVA test. For the weight, exercise capacity and mitochondrial respiratory capacity measurement a linear mixed effect model was used. Group, training and different respiration steps was set at fixed variables and animal number as random. The obtained p-values were adjusted according to the total number of tests to minimize the risk of type I errors, using the Bonferroni method. Pearson correlations were performed between MRC (complex I and II linked) and exercise performance as well as changes in exercise performance and weight. Statistical and graphical data processing were carried out in RStudio version 4.3.0 and GraphPad Prism version 9.4.1. All statistical analyses were performed with an alpha level of 0.05. The sample size for each group was *N* = 10, unless stated otherwise. The sample size was decided based on previous studies in the laboratory focusing on mitochondrial respiratory capacity (Kosik et al. 2024). Data is presented as means ± SD. On following figures one asterisk (*) indicates *p* < 0.05, two asterisks (**) indicate *p* < 0.01, three asterisks (***) indicate *p* < 0.001 and four asterisks (****) indicate *p* < 0.0001.

## Results

### Body weight and exercise capacity test

**Fig. 1:**
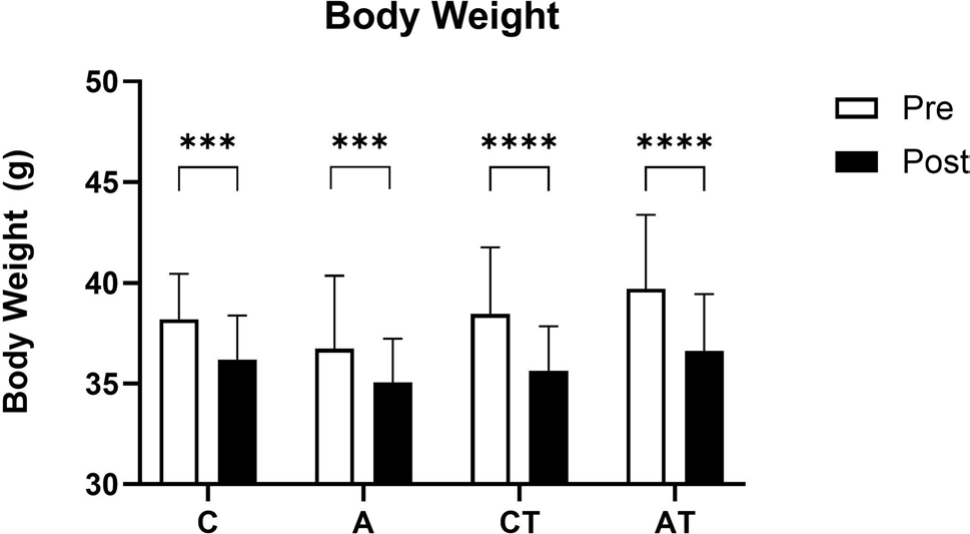
Body weight before and after the 14 days of HIIT. *N* = 10 for all groups. Values are mean ± SD. * *p* < 0.05; ** *p* < 0.01; *** *p* < 0.001; **** *p* < 0.0001. Abbreviations: A: Actovegin; AT: Actovegin and training; C: Control; CT: Control and training

**Fig. 2:**
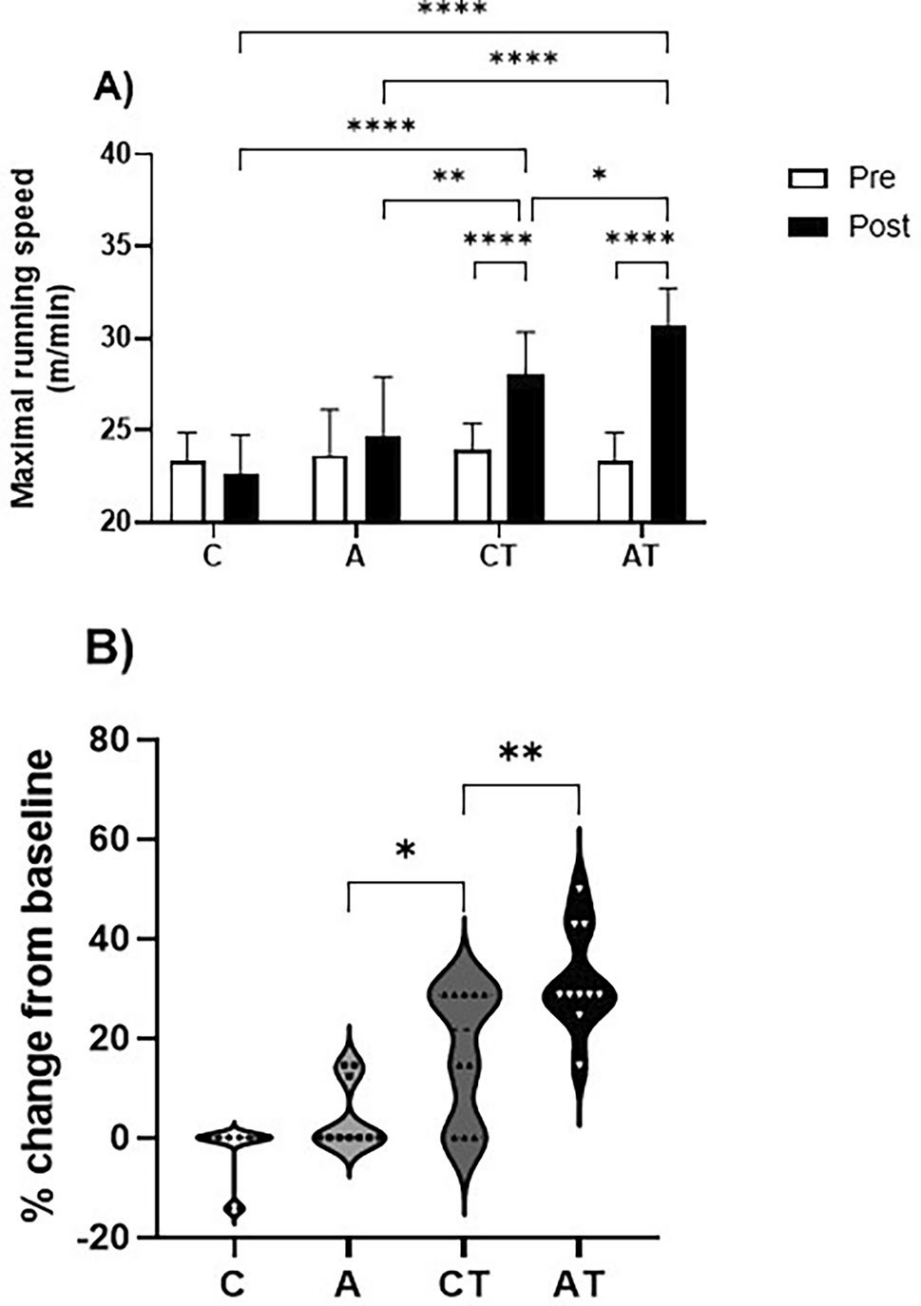
(A) Exercise performance measured as maximal running speed before and after the 14 days of HIIT. (B) Percent change in maximal running speed from baseline to post intervention shown in a violin plot. *N* = 10 for all groups. Values are mean ± SD in figure A. * *p* < 0.05; ** *p* < 0.01; *** *p* < 0.001; **** *p* < 0.0001. Abbreviations: A: Actovegin; AT: Actovegin and training; C: Control; CT: Control and training

All group lost weight during the intervention, but no differences were present between the groups (Fig. 1). Analysis of the exercise capacity test revealed a significant main effect of both administration (*p* < 0.001) and training (*p* < 0.0001) on running speed, indicating that exercise performance was increased across groups. No significant differences between groups were observed in pre-intervention running speed. Pairwise comparisons revealed significant pre to post improvements in exercise capacity for both the CT and AT groups following the intervention (17% increase, *p* < 0.0001 for CT and 32% increase, *p* < 0.0001 for AT). The CT group (pre: 24.0 ± 1.4 m/min; post: 28.0 ± 2.3 m/min) displayed a significantly higher increase in running speed than the C (pre: 23.3 ± 1.6 m/min; post: 22.7 ± 2.1 m/min) and A (pre: 23.7 ± 2.5 m/min; post: 24.7 ± 3.2 m/min) groups (*p* < 0.001 and *p* < 0.05, respectively), while the AT group (pre: 23.3 ± 1.6 m/min; post: 30.6 ± 2.1 m/min) demonstrated a higher increase in running speed compared to all other groups (*p* < 0.01) (Fig. 2A *& B*). When looking at running distance during the exercise test a similar picture was seen as in with the exercise capacity test (data not shown). There were no differences between the four groups at baseline (C: 100 ± 12 m; A: 103 ± 19 m; CT: 105 ± 11 m; AT: 100 ± 12 m). The CT group (pre: 105 ± 11 m; post: 139 ± 20 m) increased running distance compared to the C (pre: 100 ± 11 m; post: 96 ± 15 m) and A group (pre: 103 ± 19 m; post: 112 ± 25 m) (*p* < 0.0001 and *p* < 0.005, respectively), while the AT group (pre: 100 ± 12 m; post: 164 ± 20 m) demonstrated a longer running distance compared with all other groups (*p* < 0.01).

### Mitochondrial respiratory capacity

**Fig. 3:**
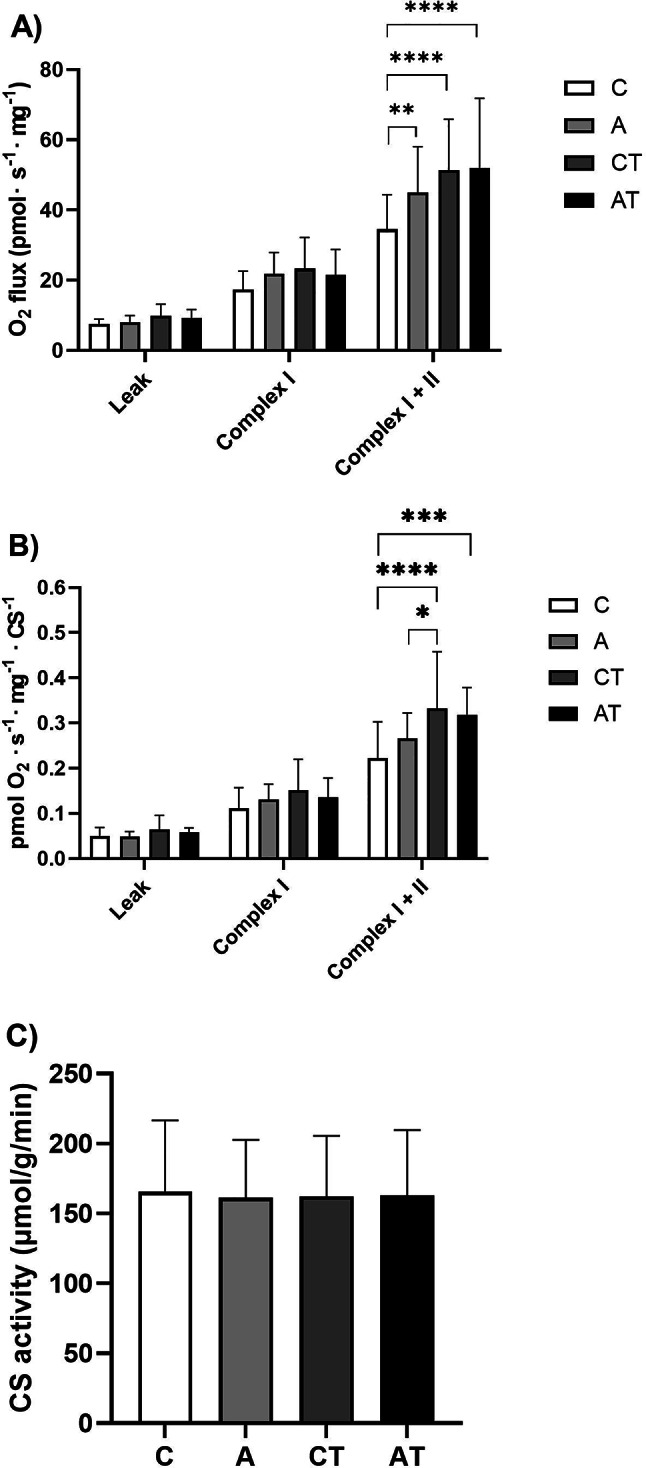
(A) Mitochondrial respiratory capacity in permeabilized muscle fibers. Leak respiration measured with malate and glutamate. Complex I linked respiration measured with malate, glutamate and ADP. Complex I + II linked respiration measured with malate, glutamate, ADP and succinate. (B) Mitochondrial respiratory capacity normalized to citrate synthase activity. *N* = 10 for the C, CT and AT groups. *N* = 8 for the A group due to unsuccessful CS measurements. (C) Citrate synthase activity in skeletal muscle. *N* = 10 for the C, CT and AT groups. *N* = 8 for the A group due to unsuccessful CS measurements. Values are mean ± SD. * *p* < 0.05; ** *p* < 0.01; *** *p* < 0.001; **** *p* < 0.0001. Abbreviations: A: Actovegin; AT: Actovegin and training; C: Control; CT: Control and training

The statistical analysis revealed that the A group displayed a 30% increase (*p* < 0.01) in MRC measured as complex I + II linked respiration compared to the C group. The CT group displayed a 49% higher (*p* < 0.0001) MRC while the AT group displayed a 51% increase (*p* < 0.0001) in MRC compared to the C group (Fig. 3A). No significant differences were observed for leak or complex I linked respiration.

No significant differences were observed between groups regarding CS activity (Fig. 3C). When normalizing MRC to CS activity, the CT group displayed a significantly higher CS activity specific flux than both the C and A group (*p* < 0.0001 and *p* < 0.05, respectively). Also, the AT group demonstrated a significantly higher CS activity specific flux than the C group (*p* < 0.001) (Fig. 3B).

## Discussion

Major findings in this study firstly include that Actovegin with HIT increases exercise performance more than HIT alone, demonstrated by the AT group displaying a larger increase in exercise capacity than all other groups. Secondly, these improvements were accompanied by an increase in MRC for the CT and AT groups compared to the C group. Furthermore, an increase was observed in MRC for the A group, indicating an isolated effect of Actovegin on MRC without exercise training. To the best of our knowledge, this is the first study demonstrating performance enhancing properties of Actovegin, as well as an increase in muscle fiber MRC with in vivo administration in healthy mice.

### Improvements in exercise capacity

As the observed increases in exercise performance were accompanied by increases in mitochondrial respiratory capacity (complex I and II linked respiration), it is relevant to determine to which degree these two factors are correlated. The analysis demonstrated no correlation between the two factors (Pearson correlation, R^2^ = 0.054, data not shown). While other studies have found strong correlation between MRC and exercise performance and improvements in time trial performance and MRC after a training intervention (Jacobs et al. 2011, 2013b), it is possible that due to the exercise test being of shorter duration and higher intensity in the present study, it is limited by mitochondrial capacity to a lesser degree than exercise tests chosen in other studies. In the present study both trained groups followed the same HIT protocol, and no differences were observed in the exercise training intensities between the CT and AT groups (84.0% and 85.7% of maximal running speed, respectively). Also, no differences were observed between groups in pre intervention exercise capacity. As such, the observed differences in post intervention exercise performance between the CT and AT groups are most likely a result of the Actovegin administration. During the intervention, all groups presented a significant reduction in body weight. Weight loss was uniform across groups and is a common response for mice being transferred from grouped to individual housing, as they were in the present study upon inclusion (Bartolomucci et al. 2003), but could also be due to the stress induced by the IP injections. A linear regression between observed weight loss and the observed changes in exercise performance showed no correlation (Pearson correlation, R^2^ = 0.042, data not shown), indicating that the loss of body weight observed in the present study cannot explain the increases seen in exercise performance. It should be mentioned that the present study is the first to report improved exercise performance after Actovegin administration. Earlier studies have looked at recovery time after muscle strains or injuries, where it has been reported that Actovegin improves recovery (Lee et al. 2011b). Other studies have investigated the acute effect of Actovegin administration on MRC (Sondergard et al. 2016) and exercise capacity (Lee et al. 2012). Sondergard and colleagues found that acute ex vivo incubation of human skeletal muscle with Actovegin increased mitochondrial respiratory capacity in a dose depended manner (Sondergard et al. 2016), which is in line with the present study. Lee and colleagues saw no differences in exercise capacity in humans after acute intravenous injection with Actovegin (Lee et al. 2012).

### Mitochondrial adaptations

A significant increase was observed in complex I + II linked MRC for both the A, CT and AT groups compared to the C group, showing that MRC can be improved in mice with just 14 days of Actovegin administration, HIT, or a combination of both. Increases in MRC following HIT similar to our findings have been reported in humans before (Jacobs et al. 2013b). One former study has also reported increases in human muscle fiber MRC with Actovegin, although Actovegin was administered acutely ex vivo and in large concentrations (Sondergard et al. 2016). Another study reported increased mitochondrial respiration (complex I and complex I + II linked oxidative phosphorylation capacity) in Actovegin treated type 1 diabetic mice skeletal muscle compared to saline treated controls, indicating Actovegin may be an effective agent for attenuating mitochondrial dysfunction associated with type 1 diabetes (Kosik et al. 2024). In the present study CS activity measurements resulted in no differences between groups, indicating that neither Actovegin administration nor 14 days of HIT is sufficient to induce changes in CS activity in mice. Similar results have previously been shown in human studies, reporting no changes in CS activity following periods of HIT (Granata et al. 2016; Gorostiaga et al. 1991). However, the findings regarding CS activity and HIT are generally contrasting, as other studies do report increases in CS activity following HIT in humans (Burgomaster et al. 2008; Gillen et al. 2016; MacInnis et al., 2017) as well as in animals (Bishop et al. 2014). As such, the relation between HIIT and mitochondrial content is yet to be fully understood. It must be mentioned that CS activity is used as a biomarker for mitochondrial content and that it has been reported to correlate well with mitochondrial volume density in a small cohort of young healthy male participants (Larsen et al. 2012). There is still a need for research on this topic, and whether similar results are seen in animal models and after different interventions (exercise, diet, etc.). As CS activity was uniform across all four groups in the present study, the increases seen in MRC in the A, CT and AT groups can be attributed to an increase in mitochondrial intrinsic function, supported by our findings on CS activity specific flux. The changes observed in MRC and mitochondria specific respiratory capacity may be the result of adaptations and regulation of proteins that could be altered independently of CS activity. Such intrinsic adaptations could include increases in mitochondrial cristae density, which has been shown to be increased in trained populations compared to untrained populations (Nielsen et al. 2017). Improved cristae density may allow for a higher volume-specific inner mitochondrial surface area and thus potentially a higher capacity for OXPHOS. Schytz and colleagues investigated mitochondrial cristae- and volume density as well as mass specific MRC in untrained, recreational active and elite runners and cross-country skiers (Schytz et al. 2024). In this study they found higher mitochondrial volume density as well as cristae density in the trained groups compared with the untrained, this was accompanied by increase mass specific MRC in the trained groups (Schytz et al. 2024). Other authors have hypothesized that increases observed in MRC and mitochondrial intrinsic function may be explained by the formation of mitochondrial supercomplexes, in which existing mitochondrial complexes can be reorganized in larger supercomplexes, allowing for improved mitochondrial respiration (Bianchi et al. 2004). The formation of supercomplexes has previously been shown to increase with exercise training in both humans (Greggio et al. 2017) and rodents (Han et al. 2022), why it may be a plausible explanation for the results observed in the present study as well.

### Diverse effects of Actovegin

Due to the many physiological components in Actovegin it is difficult to identify the precise mechanisms of action. The content of succinate in Actovegin has been thought to play a possible role in relation to the effects on mitochondria, as succinate is a potent stimulator of mitochondrial respiration. In a study by Søndergård et al. an increase in MRC was observed with acute Actovegin incubation (Sondergard et al. 2016). The authors controlled for the effects of succinate in Actovegin by running concurrent measurements with succinate concentrations corresponding to the contents in Actovegin. While they did see an increase in MRC with succinate addition, the increase was of a lower magnitude than with Actovegin. As such, they concluded that the content of succinate may explain some of the effects of Actovegin on mitochondria, but not all (Sondergard et al. 2016). It is important to note that the experiments done by Søndergård et al. were conducted with acute Actovegin incubation and direct addition of Actovegin to the oxygraph chambers. In the present study Actovegin was administered in vivo, and no additional Actovegin was added to the tissue postmortem. As such, the levels of Actovegin derived succinate were much lower in the present study than it was in the study done by Søndergård. Other authors have stated that membrane stabilizing effects of Actovegin could explain the increases in MRC observed in experiments using saponin to permeabilize cell membranes (Brock et al. 2020). It is true that too much exposure to saponin may result in harmful cell membrane damages (Bottger and Melzig 2013). However, the permeabilization of cell membranes is a prerequisite for the substrates added during titration protocols to be able to affect the mitochondria within cells (Pesta and Gnaiger 2012). The duration of saponin exposure has been shown to affect mitochondrial respiration, with 30 min of saponin exposure resulting in maximal respiratory rate compared to 0, 15–45 min (Tonkonogi et al. 1998). In the present study cell membrane integrity was controlled using cytochrome C during the titration protocols. No samples responded to cytochrome C, indicating no harmful effects of the saponin exposure. As such, the potential membrane stabilizing effects of Actovegin is highly unlikely to have influenced mitochondrial measurements in the present study. Besides its effects on mitochondria, it is also possible that Actovegin could affect other physiological parameters related to exercise capacity, including blood volume, hemoglobin mass, capillarization or functional properties of the skeletal or cardiac muscle. However, no such measurements were carried out in the present study.

Results obtained from mice in the present study are very interesting but needs to be tested in humans as well to make sure that it is relevant in humans.

The present study has several limitations. Most notably, the generalizability of the findings to human populations remains uncertain. Another thing is if the dose of Actovegin administration can be applied to humans as well. Due to limitation in personnel and available resources at the time of data collection, blinding was not feasible. The project was conducted within a small research team in which the same individuals who administered the interventions were also responsible for collecting and processing the data. With no additional staff available to serve as independent assessors, implementing a blinded design was not logistically possible.

Finally, it would have been nice to have measured performance in a sub maximal test, that would have been longer and less intensive.

## Conclusion

This is the first study to demonstrate ergogenic effects (exercise tolerance (running speed and distance) and mitochondrial respiratory capacity) of Actovegin in a preclinical mice model. Exercise capacity was increased in the CT group as a result of 14 days of HIT compared to both C and A, but was further improved in the AT group, demonstrating an additive effect of Actovegin administration with HIIT. Complex I + II linked MRC was increased in mice skeletal muscle in the A, CT and AT group, showing an isolated effect of both HIT and Actovegin administration on mitochondrial respiration. No additive effect was seen on MRC. Measurements of CS activity resulted in no differences between groups for mitochondrial content, why the increases in MRC were attributed to increased intrinsic respiratory capacity. The performance enhancing effect of Actovegin demonstrated in the present study is yet to be explained, and the transferability to humans needs to be investigated.

## Perspective

This study contributes to a better understanding of how Actovegin affects exercise capacity and mitochondrial respiratory capacity in skeletal muscle. In opposition to previous studies rejecting that Actovegin has any ergogenic effects on performance (Lee et al. 2011a; Brock et al. 2020), this study sheds new light on Actovegin as a potential performance enhancing substance. It should be acknowledged that the present study was conducted in mice, and that a direct comparison to humans requires careful conceptualization due to clear physiological and methodological differences. As this is the first study reporting such effects, further studies should aim to elaborate on these findings.

## Data Availability

All data is available from the corresponding author upon reasonable request.
